# Homeostasis model assessment of insulin resistance and outcome of ischemic stroke in non-diabetic patients - a prospective observational study

**DOI:** 10.1186/s12883-019-1406-3

**Published:** 2019-07-25

**Authors:** Daniel Åberg, N. David Åberg, Katarina Jood, Lukas Holmegaard, Petra Redfors, Christian Blomstrand, Jörgen Isgaard, Christina Jern, Johan Svensson

**Affiliations:** 1 Department of Internal Medicine, Institute of Medicine, the Sahlgrenska Academy at University of Gothenburg, Gothenburg, Sweden; 2 Center of Brain Repair and Rehabilitation, Institute of Neuroscience and Physiology, the Sahlgrenska Academy at University of Gothenburg, Gothenburg, Sweden; 3 Department of Clinical Neuroscience, Institute of Neuroscience and Physiology, the Sahlgrenska Academy at University of Gothenburg, Gothenburg, Sweden; 4 Department of Pathology and Clinical Genetics, Institute of Biomedicine, the Sahlgrenska Academy at University of Gothenburg, Gothenburg, Sweden; 5 Department of Internal Medicine, Sahlgrenska University Hospital, University of Gothenburg, Blå stråket 5, SE-413 45 Göteborg, Sweden

**Keywords:** Insulin resistance, HOMA-IR, Ischemic stroke, Modified Rankin scale

## Abstract

**Background:**

Insulin resistance (IR) in relation to diabetes is a risk factor for ischemic stroke (IS), whereas less is known about non-diabetic IR and outcome after IS.

**Methods:**

In non-diabetic IS (*n* = 441) and controls (*n* = 560) from the Sahlgrenska Academy Study on Ischemic Stroke (SAHLSIS), IR was investigated in relation to IS severity and functional outcome. IR was evaluated acutely and after 3 months using the Homeostasis model assessment of IR (HOMA-IR). Stroke severity was assessed by the National Institutes of Health Stroke Scale (NIHSS). Functional outcome was evaluated using the modified Rankin Scale (mRS) after 3 months, 2 and 7 years. Associations were evaluated by logistic regression.

**Results:**

Higher acute and 3-month HOMA-IR was observed in IS compared to the controls (both *p* < 0.001) and in severe compared to mild IS (both *p* < 0.05). High acute HOMA-IR was associated with poor outcome (mRS 3–6) after 3 months and 7 years [crude Odds ratios (ORs), 95% confidence intervals (CIs) 1.50, 1.07–2.11 and 1.59, 1.11–2.30, respectively], but not after 2 years. These associations lost significance after adjustment for all covariates including initial stroke severity. In the largest IS subtype (cryptogenic stroke), acute HOMA-IR was associated with poor outcome after 2 years also after adjustment for age and stroke severity (OR 2.86, 95% CI 1.01–8.12).

**Conclusions:**

In non-diabetic IS patients, HOMA-IR was elevated and related to stroke severity, but after adjustment for IS severity, the associations between HOMR-IR and poor outcome lost significance. This could suggest that elevated IR mostly is a part of the acute IS morbidity. However, in the subgroup of cryptogenic stroke, the associations with poor outcome withstood correction for stroke severity.

## Background

Diabetes mellitus (henceforth diabetes), type 1 as well as type 2, is associated with increased risk for ischemic stroke (IS) [1]. Insulin resistance (IR), a hallmark of diabetes type 2, may be present also in patients without manifest diabetes and is an independent primary risk factor for cardiovascular diseases (CVD) including IS [2, 3]. Furthermore, IR and hyperglycemia per se, regardless of a diagnosis of diabetes, are commonly seen in response to stressful situations such as critical illness and IS [4, 5]. In a large multicenter study (*n* = 4537), diabetes was associated with worse 3-month outcome after stroke [6]. Admission hyperglycemia was associated with poor functional outcome up to one year after IS in a study population of both diabetic and non-diabetic patients [7]. A systematic review of studies in IS (diabetics included) confirmed that stress hyperglycemia was related to poor short-term outcome [5].

In non-diabetic IS patients, hyperglycemia per se was associated with worse short-term outcome [8]. Furthermore, prediabetes tended to associate with a poor early prognosis after acute IS [9]. Also, in older non-diabetic women, homeostasis model assessment of IR (HOMA-IR), but not fasting glucose or glycated hemoglobin, was an independent risk factor of coronary heart disease and stroke [10]. In recent studies of Chinese (*n* = 173 and *n* = 1245, respectively) [11, 12] and Japanese (*n* = 4655) [13] non-diabetic patients, high HOMA-IR was associated with poor outcome up to one year after the index stroke. Therefore, in summary, diabetes and/or hyperglycemia in the acute phase of IS are known risk factors for worse outcome, but relatively few long-term studies have been performed in non-diabetic patients. We analyzed IR using the HOMA-IR method [14] in non-diabetic IS patients (*n* = 441) and controls (*n* = 560) included in the Sahlgrenska Academy Study on Ischemic Stroke (SAHLSIS) [15, 16].

## Methods

### Patients and controls

The design of SAHLSIS has previously been reported [16]. Briefly, patients with first-ever or recurrent acute IS before the age of 70 years were recruited. All patients included in the present study were enrolled consecutively at four Stroke Units in western Sweden between 1998 and 2003. The controls were randomly selected either from a population-based health survey or the Swedish Population Register, to match cases regarding age (< 1 year), sex and area of residence. Totally, there were 600 patients and 600 controls. After exclusion of all participants with diabetes, 486 patients and 560 controls were available. In the final sample, we included the 441 non-diabetic patients and 560 non-diabetic controls that had adequate blood samples for determination of HOMA-IR.

### Classification of stroke etiology

Stroke etiology was classified using the Trial of Org 10172 in Acute Stroke Treatment (TOAST) criteria [17] into the subtypes large-vessel disease (LVD), small-vessel disease (SVD), cardioembolic (CE) stroke, cryptogenic stroke (when no cause was identified despite extensive evaluation) and other determined cause of stroke. In our study, we used a local protocol as specified previously [16, 18]. According to our protocol, stroke with more than one cause was not classified as cryptogenic stroke.

### Stroke severity and functional outcome

In IS patients, initial stroke severity was originally assessed by the Scandinavian Stroke Scale (SSS). The SSS is similar to the now more commonly used National Institutes of Health Stroke Scale (NIHSS) [19], with the most important exception that the scales are inverse (i.e. higher values are beneficial in SSS). We recalculated the SSS scores to NIHSS scores using the algorithm: NIHSS = 25.68–0.43 × SSS [20]. We classified mild IS as NIHSS < 1.60 (SSS = 56–58), moderate IS as NIHSS 1.61–5.90 (SSS = 46–55), and severe IS as NIHSS > 5.90 (SSS = 1–46) [15]. Functional outcome was evaluated by the modified Rankin Scale (mRS) being graded 0–6, where 0 is no disability, 5 is severe disability, and 6 is dead. Evaluation of mRS was also performed after 2 and 7 years. The mRS score was dichotomized for death or dependency, i.e. poor functional outcome (mRS 3–6) versus favorable functional outcome (mRS 0–2).

### Assessment of covariates and ethical considerations

Anthropometric parameters (body mass index, BMI) and data on hypertension, diabetes and smoking were recorded as described previously [16]. Diabetes was defined as receiving diet treatment or medication for diabetes or alternatively, as fasting plasma glucose ≥7.0 mmol/L or fasting blood glucose ≥6.1 mmol/L for at least two occasions [21]. Among cases, measurements performed at 3-month follow-up were used for the definition of diabetes and hypertension. Hypertension was defined by pharmacological treatment for hypertension, systolic blood pressure ≥ 160 mmHg, and/or diastolic blood pressure ≥ 90 mmHg [16, 22]. BMI was calculated as kg/m^2^. All participants or their next of kin provided written informed consent. This study was approved by the Ethics Committee of the University of Gothenburg.

### Biochemical analysis

Venous blood samples were collected in the acute phase (at 1–10 days after index stroke; median 4 days), and at the 3-month follow-up in IS cases (median 101, range 85–125 days) and once in controls. Blood sampling was performed between 08:30 and 10:30 AM after overnight fasting of > 8 h. Blood glucose or plasma glucose was analyzed, and blood glucose values were transformed to plasma glucose according to the formula: plasma glucose = blood glucose × 1.11. All blood and plasma concentrations of glucose and low-density lipoprotein cholesterol (hereafter LDL) were analyzed using standardized methods at the Department of Clinical Chemistry at the Sahlgrenska University Hospital. Serum levels of insulin and C-reactive protein (CRP) were analyzed by a solid-phase chemiluminescent immunometric assay on IMMULITE 2000 (Diagnostic Products Corporation, USA) using the manufacturers reagents as directed. HOMA-IR was calculated as fasting insulin (microU/L) x fasting glucose (nmol/L) / 22.5 [14].

### Statistical evaluation

**Fig. 1:**
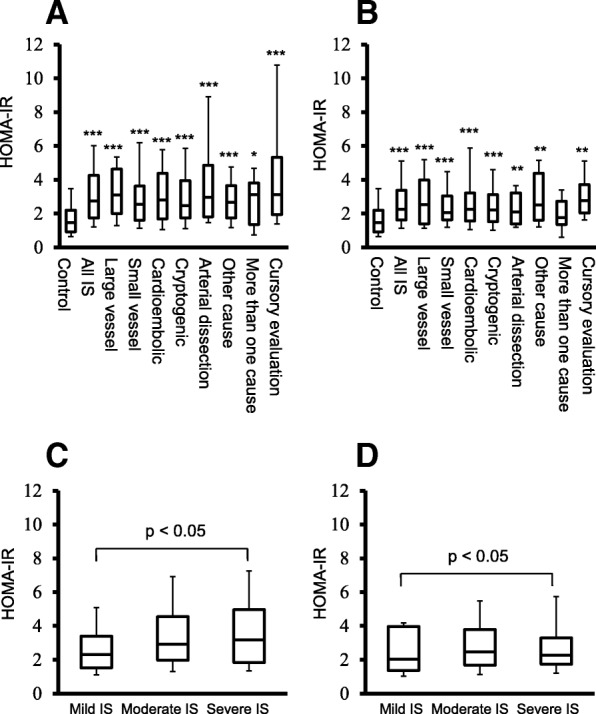
Panels **a** and **b** display HOMA-IR in the control group (*n* = 560), in all IS patients (*n* = 441), and in the IS subtypes large vessel disease (*n* = 40), small vessel disease (*n* = 91), cardioembolic stroke (*n* = 75), cryptogenic stroke (*n* = 123), arterial dissection (*n* = 28), other causes (*n* = 13), more than one cause (*n* = 17), and cursory evaluation (*n* = 54). Panel **a**) HOMA-IR in the acute phase and Panel B) HOMA-IR after 3 months. Panels **c** and **d** display HOMA-IR according to stroke severity (NIHSS score > 5.90 = severe, NIHSS 1.61–5.90 = intermediate and NIHSS ≤1.60 mild IS). Panel **c**) HOMA-IR in the acute phase and **d**) HOMA-IR after 3 months. Values in the box plots are given as medians (horizontal lines), 25th–75th percentiles (boxes), and 10th–90th percentiles (whiskers). The statistical analyses were performed using the Kruskal-Wallis test for multiple groups followed by the Mann-Whitney U-test for pairwise comparisons. *p < 0.05, **p < 0.01, ***p < 0.001 compared to controls

Between-group differences of means were analyzed using ANOVA followed by Tukey’s HSD post hoc analysis for continuous variables and using chi-square tests for categorical variables. In Fig. 1, between-group differences of medians were calculated using the Kruskal-Wallis test for multiple groups followed by the Mann-Whitney U-test for pairwise comparisons.

To evaluate whether HOMA-IR was associated with functional outcome (mRS), we calculated odds ratios (ORs) and 95% confidence intervals (CIs) using binary logistic regression analysis. In this statistical analysis, HOMA-IR was logarithmically transformed. As evaluated using the Kolmogorov-Smirnov test, HOMA-IR showed a skewed distribution prior to the logarithmical transformation, but after the logarithmical transformation, it was normally distributed. Since we intended to examine the independent effect of HOMA-IR on functional outcome, adjustments were made for predefined covariates that are related to stroke severity and well known metabolic risk factors [15, 16, 23, 24]. In Table 3, Model A included the vascular covariates age, sex, smoking, hypertension, LDL and BMI. Acute LDL was used as covariate in the model for acute HOMA-IR and 3-month LDL was used as a covariate in the model for 3-month HOMA-IR. Model B, in addition to the former covariates, also included the covariate stroke severity (NIHSS in the acute phase).

Data were analyzed using SPSS version 20.0 (SPSS Inc., Chicago, IL). *P*-values < 0.05 were considered significant.

### Missing values

In Tables 3 and 4, out of the 441 cases at the acute phase and the 429 cases after 3 months that had blood samples available for analysis of HOMA-IR, the number in the logistic regression analysis was reduced over time due to missing values of the outcome mRS. However, for the missing values of the covariates included in the logistic regression analysis, imputed dummy variables were introduced for the categorized variables and mean values replaced the missing continuous variables.

## Results

### Baseline characteristics

Baseline characteristics of SAHLSIS population have been reported previously [15, 16, 24, 25]. In the present study, we excluded all patients and controls having diabetes. Characteristics of the included non-diabetic participants are given in Table 1. As expected, the hypertension, smoking, history of coronary disease (=coronary event or angina pectoris) as well as atrial fibrillation were more common in patients. LDL levels and BMI did not significantly differ between patients and healthy controls, whereas acute CRP was higher in the patients. HOMA-IR was higher in the acute phase than after 3 months (*p* < 0.01), but at both time points, HOMA-IR was higher in IS patients compared to controls (both *p* < 0.001; Table 1). In an additional analysis, acute HOMA-IR was found to correlate positively with CRP (*p* = 0.014, r = 0.12).

**Table 1:** Baseline characteristics and HOMA-IR in patients with ischemic stroke and controls

	Cases		Controls		
	n	Mean ± SEM	n	Mean ± SEM	*p*-value
Sex (ratio m/f)	441	1.37 ± 0.02	560	1.37 ± 0.02	0.84
Age (years)	441	56.1 ± 0.51	560	56.6 ± 0.44	0.44
Hypertension (%)	438	58 ± 2.4	559	36 ± 2.0	< 0.001
Smoking (%)	440	39 ± 2.3	560	18 ± 1.6	< 0.001
Atrial fibr (%)	396	11 ± 1.5	513	1 ± 0.4	< 0.001
CAD (%)	432	11 ± 1.5	560	0 ± 0	< 0.001
BMI (kg/m^2^)	433	26.1 ± 0.20	559	26.3 ± 0.17	0.30
Acute LDL (mmol/L)	395	3.35 ± 0.05	558	3.33 ± 0.04	0.75
Acute CRP (mg/L)	433	11.4 ± 0.98	550	5.92 ± 0.22	< 0.001
Acute p-glucose	441	5.69 ± 0.06	560	5.09 ± 0.03	< 0.001
3-month glucose	429	5.21 ± 0.03	560	5.09 ± 0.03	0.005
Acute insulin	441	14.3 ± 0.63	560	8.26 ± 0.39	< 0.001
3-month insulin	429	12.1 ± 0.40	560	8.26 ± 0.39	< 0.001
Acute HOMA-IR^a^	441	2.84 ± 0.18	560	1.43 ± 0.09	< 0.001
3-month HOMA-IR^a^	429	2.34 ± 0.11	560	1.43 ± 0.09	< 0.001

If not otherwise stated, data are shown as means and ± SEM. In addition, n is shown as numbers vary due to missing data. Differences between cases and controls were examined by ANOVA. *ns* not significant, *LDL* low-density lipoprotein, *BMI* body mass index, *CRP* C-reactive protein, *CAD* History of Coronary Artery Disease before the event; Atrial fibr, Atrial Fibrillation before the event; Smoking, Current Smoking. ^a^given as geometric mean and ± SEM

### HOMA-IR, IS subtypes, and stroke severity

HOMA-IR in controls and all IS as well as IS subtypes are given in Fig. 1. HOMA-IR both in the acute phase (Fig. 1a) and after 3 months (Fig. 1b) was significantly elevated not only in all IS, but also in all of the main IS subtypes, compared to the healthy controls.

Baseline clinical data in relation to stroke severity are presented in Table 2. Acute CRP was higher in patients with severe stroke compared to those with mild stroke. HOMA-IR was higher in severe IS than in mild IS both in the acute phase and after 3 months (both *p* < 0.05; Table 2 and Fig. 1c and d).

**Table 2:** Baseline characteristics and HOMA-IR in relation to stroke severity

	Stroke severity						
	Severe		Moderate		Mild		
	n	Mean ± SEM	n	Mean ± SEM	n	Mean ± SEM	*p*-value
Sex (ratio m/f)	114	1.39 ± 0.05	167	1.37 ± 0.04	160	1.36 ± 0.04	0.59
Age (years)	114	56.1 ± 0.95	167	57.0 ± 0.80	160	55.2 ± 0.89	0.49
Hypertension (%)	112	57 ± 4.7	166	64 ± 3.7	160	52 ± 4.0	0.39
Smoking (%)	113	48 ± 4.7	167	30 ± 3.6	160	42 ± 3.9	0.34
Atrial fibr (%)	113	14 ± 3.6	149	11 ± 2.5	150	8 ± 2.2	0.11
CAD (%)	97	16 ± 3.5	167	10 ± 2.4	158	9 ± 2.3	0.071
BMI (kg/m^2^)	110	25.6 ± 0.42	166	26.7 ± 0.36	157	25.7 ± 0.29	0.86
Acute LDL (mmol/L)	98	3.22 ± 0.10	147	3.40 ± 0.08	150	3.39 ± 0.08	0.18
Acute CRP (mg/L)	113	16.4 ± 2.32	164	10.7 ± 1.77	156	8.65 ± 0.97	0.002
Acute p-glucose	114	6.14 ± 0.15	167	5.64 ± 0.09	160	5.41 ± 0.04	< 0.001
3-month p-glucose	106	5.21 ± 0.05	171	5.23 ± 0.05	152	5.17 ± 0.05	0.69
Acute insulin	114	15.2 ± 1.28	167	15.1 ± 1.10	160	12.9 ± 1.05	0.18
3-month insulin	106	12.5 ± 0.88	171	13.4 ± 0.81	152	10.5 ± 0.48	0.03
Acute HOMA-IR^a^	114	3.22 ± 0.41	167	2.95 ± 0.29	160	2.47 ± 0.28	0.02
3 month HOMA-IR^a^	106	2.48 ± 0.24	171	2.50 ± 0.21	152	2.00 ± 0.13	0.02

The patients were categorized using the acute National Institutes of Health Stroke Scale (NIHSS) as having severe (NIHSS score 5.91–25.25), moderate (NIHSS score 1.61–5.90) or mild (NIHSS stroke 0–1.60) stroke. If not otherwise stated, data are shown as means (if not otherwise stated) and ± SEM. In addition, n is shown as numbers vary due to missing data. Differences between severe and mild stroke were examined by ANOVA. *ns* not significant, *LDL* low-density lipoprotein, *BMI* body mass index, *CRP* C-reactive protein, *CAD* History of Coronary Artery Disease before the event; Atrial fibr, Atrial Fibrillation before the event; Smoking, Current Smoking. ^a^given as geometric mean and ± SEM

### HOMA-IR and functional outcome

In the total IS population, we investigated whether HOMA-IR in the acute phase or after 3 months was associated with functional outcome. High acute HOMA-IR was associated with a poor outcome (mRS 3–6) after 3 months (crude OR 1.50, 95% CI 1.07–2.11) and after 7 years (crude OR 1.59, 95% CI 1.11–2.30) (Table 3). After adjusting for cardiovascular covariates in Model A, acute HOMA-IR was still associated with a poor outcome (mRS 3–6) after 3 months (OR 1.69, 95% CI 1.16–2.46) and 7 years (OR 2.01, 95% CI 1.31–3.10). Furthermore, deaths occurring a long time after IS may not be related to the stroke. Therefore, we performed additional analyses for the 7-year outcome in which we excluded patients who had died (= mRS 6), and these analyses showed similar associations (Table 3). However, when stroke severity was added (Model B), no significant associations remained between acute HOMA-IR and functional outcome at any time point (Table 3). High HOMA-IR after 3 months did not associate with poor functional outcome (mRS 3–6) after 2 or 7 years (Table 3).

**Table 3:** Risk of poor IS outcome per unit increase in log HOMA-IR

	n	Crude	Model A	Model B
Acute HOMA-IR				
3 months	423	1.50 (1.07–2.11)*	1.69 (1.16–2.46)**	1.12 (0.60–2-06)
2 years	436	1.22 (0.86–1.71)	1.32 (0.90–1.92)	0.89 (0.51–1.55)
7 years Deaths (mRS 6) included	294	1.59 (1.11–2.30)*	2.01 (1.31–3.10)**	1.75 (0.97–3.16)^†^
7 years Deaths (mRS 6) excluded	254	1.50 (0.96–2.33)^††^	1.72 (1.03–2.87)*	1.23 (0.59–2.57)
3 months HOMA-IR				
3 months	420	1.25 (0.84–1.85)	1.53 (0.95–2.45)^‡^	1.27 (0.69–2.42)
2 years	426	1.15 (0.77–1.71)	1.24 (0.77–1.98)	1.04 (0.56–1.94)
7 years Deaths (mRS 6) included	289	0.97 (0.64–1.48)	1.03 (0.61–1.74)	0.91 (0.49–1.70)
7 years Deaths (mRS 6) excluded	257	0.93 (0.56–1.55)	0.80 (0.43–1.50)	0.70 (0.31–1.58)

Footnote: Odds ratios (ORs) and 95% confidence intervals (CIs) of having a poor outcome (mRS ≥3) at different time points after IS stroke per unit increase in log-HOMA-IR. Adjusted ORs for covariates. Model A: age, sex, BMI, hypertension, LDL and smoking. Model B: age, sex, BMI, hypertension, LDL, smoking and stroke severity (acute NIHSS). ^†^*p* = 0.06, ^††^*p* = 0.07, ^‡^*p* = 0.08 *p < 0.05, **p < 0.01

### HOMA-IR and functional outcome in stroke subtypes

Crude associations between acute HOMA-IR and outcome in the four largest subtypes of IS according to the TOAST criteria of etiology are presented in Table 4. Of note is that in the IS subtype with the largest number of subjects, cryptogenic stroke, high acute HOMA-IR was more robustly associated with poor functional outcome after 3 months (crude OR 3.65, 95% CI 1.53–8.72; *p* < 0.01) and 2 years (crude OR 4.77, 95% CI 1.93–11.8; *p* < 0.001). This contrasted to lower, non-significant ORs in the other subtypes. Due to the low number of patients in each IS subtype, we found it proper to adjust for covariates only in this largest subtype, i.e. cryptogenic stroke (numbers with poor outcome: *n* = 19 at 3 months, *n* = 19 at 2 years, *n* = 17 at 7 years and *n* = 8 at 7 years excluding deaths), and to restrict the number of covariates to two. After adjustment for the two covariates with the largest influence on outcome (age and acute NIHSS), the association between acute HOMA-IR and 3-month outcome lost significance (adjusted OR 1.66, 95% CI 0.50–5.44, data not shown), whereas the association with 2-year outcome remained (adjusted OR 2.89, 95% CI 1.02–8.20; *p* = 0.047, data not shown). There was no statistically significant association between acute HOMA-IR and 7-year outcome, either with or without exclusion of mRS 6, in any IS subtype (Table 4).

**Table 4:** Risk of poor outcome in IS subtypes per unit increase in acute log HOMA-IR

	n	Crude OR
3 months		
Large vessel disease	37	0.73 (0.19–2.85)
Small vessel disease	85	0.31 (0.07–1.30)
Cardioembolic stroke	74	1.81 (0.85–3.85)
Cryptogenic stroke	119	3.65 (1.53–8.72)**
2 years		
Large vessel disease	40	0.39 (0.10–1.47)
Small vessel disease	90	0.37 (0.11–1.20)
Cardioembolic stroke	74	1.29 (0.61–2.73)
Cryptogenic stroke	122	4.77 (1.93–11.8)***
7 years Deaths (mRS 6) included		
Large vessel disease	30	2.36 (0.59–9.45)
Small vessel disease	61	0.92 (0.30–2.77)
Cardioembolic stroke	51	1.55 (0.70–3.42)
Cryptogenic stroke	93	2.09 (0.90–4.87)^†^
7 years Deaths (mRS 6) excluded		
Large vessel disease	22	0.76 (0.14–4.03)
Small vessel disease	59	1.16 (0.36–3.77)
Cardioembolic stroke	35	1.25 (0.42–3.77)
Cryptogenic stroke	84	2.30 (0.74–7.16)

Odds ratios (ORs) and 95% confidence intervals (CIs) of having a poor outcome (mRS ≥3) for the main etiologies of IS (according to TOAST) at different time points per unit increase in acute log-HOMA-IR. ^†^*p* = 0.08, **p < 0.01, ***p < 0.001

HOMA-IR after 3 months did not associate with outcome after 2 or 7 years in any IS subtype (data not shown).

## Discussion

In this study of well-characterized non-diabetic IS patients, we found that HOMA-IR declined between the acute phase and the 3-month follow-up. However, at both time points, HOMA-IR was higher in IS patients than in the population-based non-diabetic controls, and this was true for all main etiological subtypes of IS. Furthermore, HOMA-IR was higher in severe than in mild IS at both time points. Acute HOMA-IR was associated with poor functional outcome (mRS 3–6) after 3 months and 7 years, but these associations abided after adjustment for stroke severity. However, in cryptogenic stroke (the most common IS subtype), the association between high acute HOMA-IR and poor outcome after 2 years was independent of age and stroke severity. In contrast, HOMA-IR after 3 months did not associate with functional outcome at any time point of follow-up.

To the best of our knowledge, this is the first study to investigate HOMA-IR in relation to both stroke severity and long-term outcome in a large cohort of non-diabetic Caucasian patients with IS. We found that both acute and 3-month HOMA-IR were higher in severe compared to mild stroke. Although hyperglycemia has been found to be common after IS [1, 5, 26], the association between IR and stroke severity in non-diabetic patients has been less investigated and with different findings. In the study by Calleja et al., in which insulin treatment was an exclusion criteria, NIHSS score did not differ between HOMA-IR tertiles (*n* = 109) [27], while Bas et al. found that non-diabetic IS patients with admission HOMA-IR > 2.7 had marginally increased NIHSS score compared to those with HOMA-IR < 2.7 (*n* = 180) [28]. The present relatively large study (*n* = 441) therefore establishes that IR is related to stroke severity also in non-diabetic IS patients.

In the present study, high acute HOMA-IR was associated with poor functional outcome 3 months and 7 years after the index stroke. These association between high acute HOMA-IR and poor functional outcome remained after adjustment for cerebrovascular risk factors (Model A), but did not withstand adjustment for both vascular confounding factors and initial stroke severity (Model B). Thus, when assuming IR is not a mediator of ischemic injury, but rather secondary to the injury and thereby adding initial stroke severity as a covariate, the associations disappeared. In previous studies, poor functional outcome up to one year after IS was observed in patients with hyperglycemia and/or diabetes [6–8, 29, 30]. Furthermore, hyperglycemia was related to increased short- (30 days) and long-term (1 and 6 year) mortality in an IS population including diabetic patients [31]. Baird et al. [32] found that prolonged poststroke hyperglycemia was independently associated to poor short-term clinical outcome as well as infarct expansion. Possibly, the effects of persistent hyperglycemia in the penumbra of IS might be detrimental, resulting in reduced penumbral salvage, larger final infarct size, and worse clinical outcome [33]. In Chinese (*n* = 173; non-diabetics) [11] and Japanese (*n* = 4655; no insulin treatment) [13] patients, high HOMA-IR index was associated with poor short-term functional outcome (mRS at discharge and 3 months, respectively). Finally, in a Chinese non-diabetic IS cohort (*n* = 1245) followed up to one year after IS, HOMA-IR in the acute phase was associated with increases in the risks of death, stroke recurrence and poor outcome (mRS 3–6), but not with dependence (mRS 3–5) [12]. However, there is no previous long-term study in a non-diabetic Caucasian IS population, and the importance of HOMA-IR could be different in Asian and Caucasian patients [34, 35].

There are a few studies that might implicate different mechanisms of how IR is unfavorable after IS. Two smaller studies (*n* = 109 and *n* = 180) specifically investigated the association between HOMA-IR and 3-month functional outcome in non-diabetic IS patients that had received intravenous thrombolysis, and an independent association between high HOMA-IR and poor outcome was observed [27, 28]. In a large register study of IS patients treated with intravenous thrombolysis (*n* = 16049), high admission blood glucose was associated with an increased risk of symptomatic intracerebral hemorrhage, higher mortality, and poor functional outcome as assessed by mRS after 3 months [36]. During the inclusion period for the present study, very few patients received thrombolytic treatment in our region, and among the patients studied here only 3 patients received thrombolysis. Therefore, intracerebral hemorrhage due to thrombolysis is not a relevant mechanism to explain our association between HOMA-IR and long-term outcome of IS.

Acute illness, including IS, is associated with stress hyperglycemia and increased IR [4, 5, 37]. In line with this, we observed that HOMA-IR was higher in the acute phase of IS than after 3 months. In addition, we found a positive correlation between HOMA-IR in the acute phase and acute CRP. Possibly, acutely elevated IR can be seen as part of the acute IS morbidity whereas IR after 3 months more reflects IR under steady state conditions. In contrast to acute HOMA-IR, no associations to outcome were observed for 3-month HOMA-IR. It may be speculated that as most neurological recovery occurs early after the ischemic event, IR in the acute phase has a larger impact on stroke recovery and thus on outcome than IR 3 months after the event. On the other hand, IR is a principle component of the metabolic syndrome along with central obesity, hypertension, and dyslipidemia, which contributes to bad outcome in many different contexts [38, 39]. Therefore, behavioral, lifestyle and/or therapeutic interventions that improve glucose homeostasis could also reduce metabolic derangements and potentially improve IS outcome. In the IRIS trial, which included non-diabetic patients who had insulin resistance and a recent history of IS or transient ischemic attack (TIA), the risk of stroke or myocardial infarction was lower in patients receiving pioglitazone treatment [40].

We found that HOMA-IR was elevated both in the acute phase and after three months in all main IS subtypes, i.e. large vessel disease, small vessel disease, cardioembolic stroke and cryptogenic stroke compared to the controls, suggesting that IR is a common feature of IS regardless of IS subtype. In the largest subtype that represents about one quarter of IS cases in the age group under study here, i.e. cryptogenic stroke, we observed a significant association between high acute HOMA-IR and poor outcome after 3 months and 2 years. Importantly, this association remained after adjustment for age and stroke severity. By definition, there is no obvious cause of IS in cryptogenic stroke [41], but infections/inflammation and hypercoagulability have been proposed as underlying mechanisms [42]. Although highly speculative, in the absence of obvious underlying mechanisms such as atherosclerosis or atrial fibrillation in cryptogenic stroke, the relative role of acute IR for outcome could be more pronounced. However, the association between acute HOMA-IR and 2-year outcome in cryptogenic stroke must be considered as a preliminary observation until confirmed in future studies having this as a primary hypothesis.

### Strengths and limitations

Our study included a relatively large sample of consecutive and well-characterized IS patients and population-based controls, and few patients were lost to follow-up. Only Caucasian participants were studied, which might limit the generalizability of our results, but could be of importance [34, 35] when comparing our results with those of studies with Asian origin [11–13]. Our patients were classified into etiological subtypes of IS, but despite the relatively large study sample, we did not have enough power to adjust for stroke severity and other possible confounders of outcome in all subtypes. Furthermore, the relatively young mean age of the participants (56 years) could have favored the inclusion of less severe IS cases with low fatality, and to some degree disfavored the inclusion of older patients with IS due to cardiovascular causes. We estimated IR using the HOMA-IR, which is commonly used in clinical studies [43], and there is a high correlation between IR estimations using HOMA-IR and the euglycemic clamp method [14]. An advantage of HOMA-IR compared to OGTT is that it can be performed in the initial acute phase of IS, and in severe strokes with for instance unconsciousness or dysphagia, situations in which OGTT may be detrimental and thus is contraindicated. Treatment of diabetes, e.g. insulin, could interfere with HOMA-IR calculations, but we excluded all diabetic patients and controls. However, we do not have full record of the on occasion medicine, and we therefore cannot rule out that a few patients could have received insulin injections in the acute phase of IS. Also, we do not have record of all other medications that could affect glucose homeostasis, e.g. thiazides. Another limitation is that blood samples usually were not taken directly after falling ill in IS; they were taken at 1–10 days (median 4 days), hence to a large extent reflecting stress hyperglycemia. However, if CRP, which more than NIHSS may reflect the acute stress response, was added to the binary logistic regression model, this did not change the associations exhibited in Table 3 (data not shown).

## Conclusions

Our study shows that HOMA-IR is elevated both in the acute phase of IS and at the follow-up after 3 months, and that HOMA-IR associates with worse stroke severity in non-diabetic IS patients. Acute HOMA-IR, but not HOMA-IR at 3 months, was associated with worse functional outcome after 3 months and 7 years, but these associations became weaker and non-significant after adjustment for stroke severity. Possibly, this could mean that elevated HOMA-IR is part of the acute morbidity in IS and that the prognostic value of acute HOMA-IR is primarily linked to that of other negative prognostic factors including initial stroke severity. Finally, in the largest IS subtype cryptogenic stroke, we observed an association between acute HOMA-IR and poor outcome after 2 years that remained significant after adjustment for age and stroke severity. This indicates the IR may be a relatively more important factor in cryptogenic stroke than in other stroke subtypes, but the physiological relevance of this association needs to be explored in further studies.

## Data Availability

The datasets used and/or analyzed during the current study are available from the corresponding author upon reasonable request.

## Abbreviations

CI: 95% confidence interval
HOMA-IR: The Homeostasis model assessment of IR
IR: Insulin resistance
IS: Ischemic stroke
mRS: modified Rankin Scale
NIHSS: National Institutes of Health Stroke Scale
SSS: Scandinavian Stroke Scale
TOAST: Trial of Org 10172 in Acute Stroke Treatment
